# Improved separation of rare earth elements using hydrophobic deep eutectic solvents: liquid–liquid extraction to selective dissolution†

**DOI:** 10.1039/d4gc01089b

**Published:** 2024-07-11

**Authors:** Takafumi Hanada, Nicolas Schaeffer, Masahiro Katoh, Joao A. P. Coutinho, Masahiro Goto

**Affiliations:** a Department of Applied Chemistry, Graduate School of Technology, Industrial and Social Sciences, Tokushima University 2-1 Minamijosanjima Tokushima Japan hanada@tokushima-u.ac.jp; b CICECO-Aveiro Institute of Materials, Department of Chemistry, University of Aveiro 3810-193 Aveiro Portugal; c Department of Applied Chemistry, Graduate School of Engineering, Kyushu University 744 Motooka Fukuoka Japan goto.masahiro.651@m.kyushu-u.ac.jp

## Abstract

Deep eutectic solvents (DESs) composed of a beta-diketone and phosphine oxides featuring different alkyl chain lengths were prepared and then employed as liquid–liquid extraction and dissolution media for the separation of iron, cobalt, neodymium, and dysprosium. In the dissolution the more sterically hindered DESs exhibited enhanced selectivity to neodymium, which was poorly separated using liquid–liquid extraction. The dissolution selectivity in less sterically hindered DESs could be controlled by the addition of organic acids with different alkyl chain lengths.

## Abstract 2

Tuning steric environment of hydrophobic deep eutectic solvents improves dissolution selectivity of rare earth elements.

## Graphical abstract

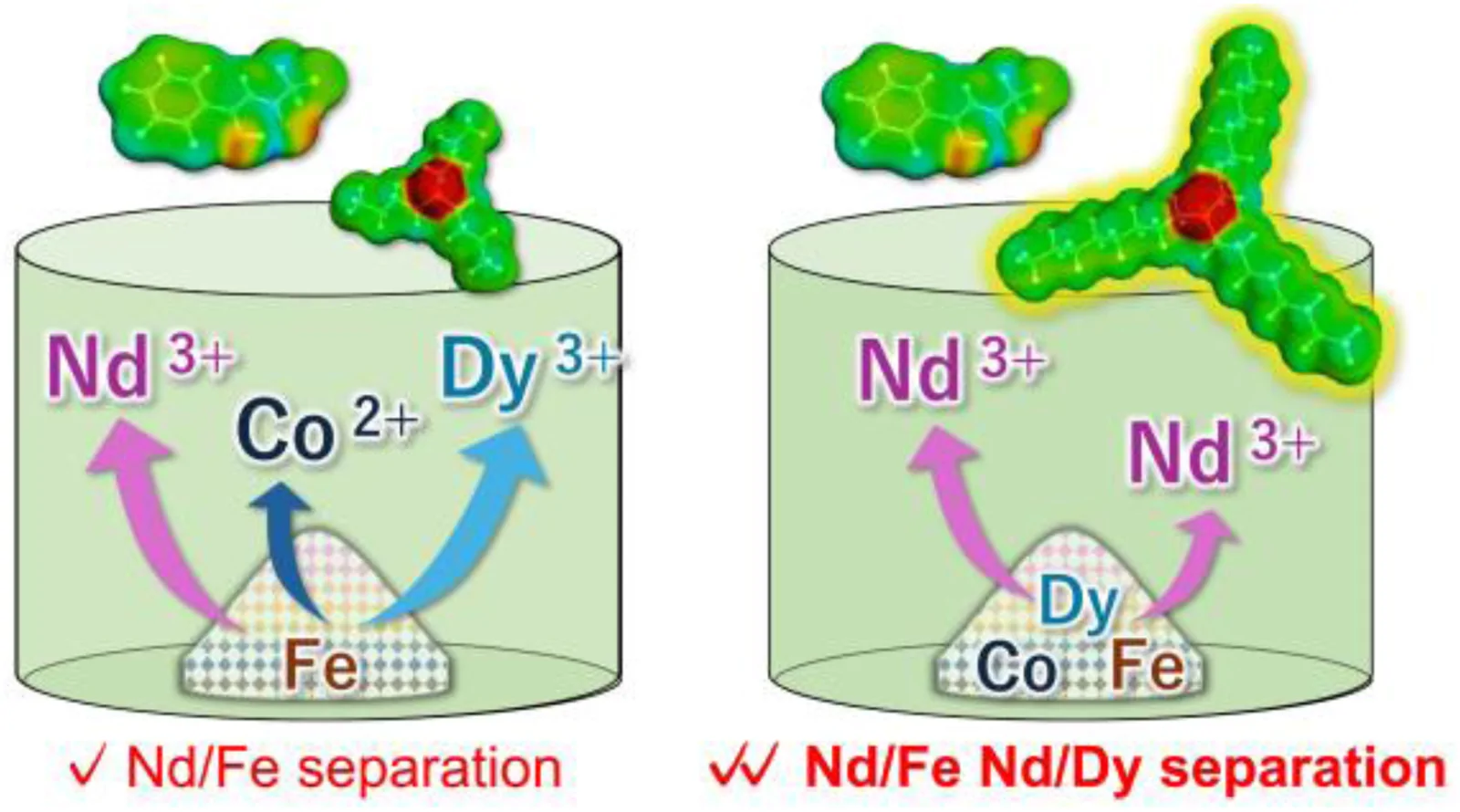

The growing consumption of rare earth elements, needed for the rapid development of renewable energy technologies, is expected to lead to serious supply risks and environmental costs associated with unsustainable mining practices.^1^ The recycling of end-of-life products, such as rare earth permanent magnets, not only reduces these risks but also minimizes the environmental destruction and pollution associated with the extraction of primary resources. However, the recycling rate of rare earths remains inadequately low at less than 1%.^1^

The recycling techniques developed for rare earths are classified as pyrometallurgical or hydrometallurgical processes.^2^ A typical hydrometallurgical route involves acid leaching, with inorganic acids used to dissolve various metals into the aqueous phase, followed by liquid–liquid extraction to separate rare earth ions. However, the mutual separation of rare earths by extraction requires large mixer–settler plant with hundreds of extraction stages due to the similar ionic properties of the different rare earths.^3^ Moreover, the inorganic acids and organic solvents used in conventional hydrometallurgical recycling are associated with environmental pollution emissions. Therefore, greener processes that enable selective and efficient rare earth recovery must be developed.

The use of functional solvents, such as ionic liquids (ILs) and deep eutectic solvents (DESs), is attracting attention as alternative media for metal processing, both for metal dissolution and their separation by solvent extraction.^4–7^ Metal dissolution in hydrophilic DESs was investigated from the beginning of DES exploration owing to its distinctive solubilization performance.^8–11^ Rare earth dissolution into DESs was achieved from model metal systems as well as real waste.^12–30^ However, knowledge of the molecular design principle of DESs to allow for tuning the metal dissolution selectivity is currently missing.^31^ Additionally, the high aqueous solubility of most DES components traditionally used in metal leaching precludes their use for the solvent extraction separation of rare earths. Hydrophobic DESs, which form two phases with water, were studied as solvent extraction media over the past decade,^32,33^ with the number of reports regarding hydrophobic DESs for rare earth extraction increasing rapidly in recent years.^34–42^ However, minimal attention has been paid to hydrophobic DESs as dissolution media for metal species, although they allow extensive molecular design for more selective and efficient metal recovery.^43^ The ease of metal recovery from hydrophobic DESs ensures the DESs’ reusability, making the entire process more sustainable.^44^

In this study, we investigate the impact of the steric hindrance of hydrogen bond acceptors (HBAs) in hydrophobic DESs on the liquid–liquid extraction and dissolution behavior of metal oxides. DESs with the same coordination group but different steric environments were prepared. Cobalt (Co), iron (Fe), neodymium (Nd), and dysprosium (Dy) were employed to facilitate the study of the separation behavior of metal oxides with different charges and ionic radii. This is the first report of a dissolution behavior of rare earths in hydrophobic DESs.

First, two DESs composed of a beta-diketone and a phosphine oxide were prepared (Fig. 1). The combination of a beta-diketone and a phosphine oxide provides a synergistic enhancement of alkali and transition metal ions during solvent extraction.^45^ Here, benzoyltrifluoroacetone (HBTA) as a hydrogen bond donor (HBD) and trioctylphosphine oxide (TOPO) or tributylphosphine oxide (TBPO) as HBAs were employed to prepare DESs. Because these two HBAs have different alkyl chain lengths but the same coordination group, only the steric environment of the DESs was varied. We successfully prepared the DESs using a molar ratio of HBD : HBA = 2 : 1. The physical properties of the DESs are shown in Table S1.† The DESs have low viscosity (<15 mPa s), which is advantageous in mass transfer. There is an increase of the viscosity after the addition of water, probably due to the recombination of the hydrogen bonding network. Because the DESs were hydrophobic enough to form two phases with water, we could assess the DESs’ selectivity for a given ion when applied to either liquid–liquid extraction or dissolution.

**Fig. 1:**
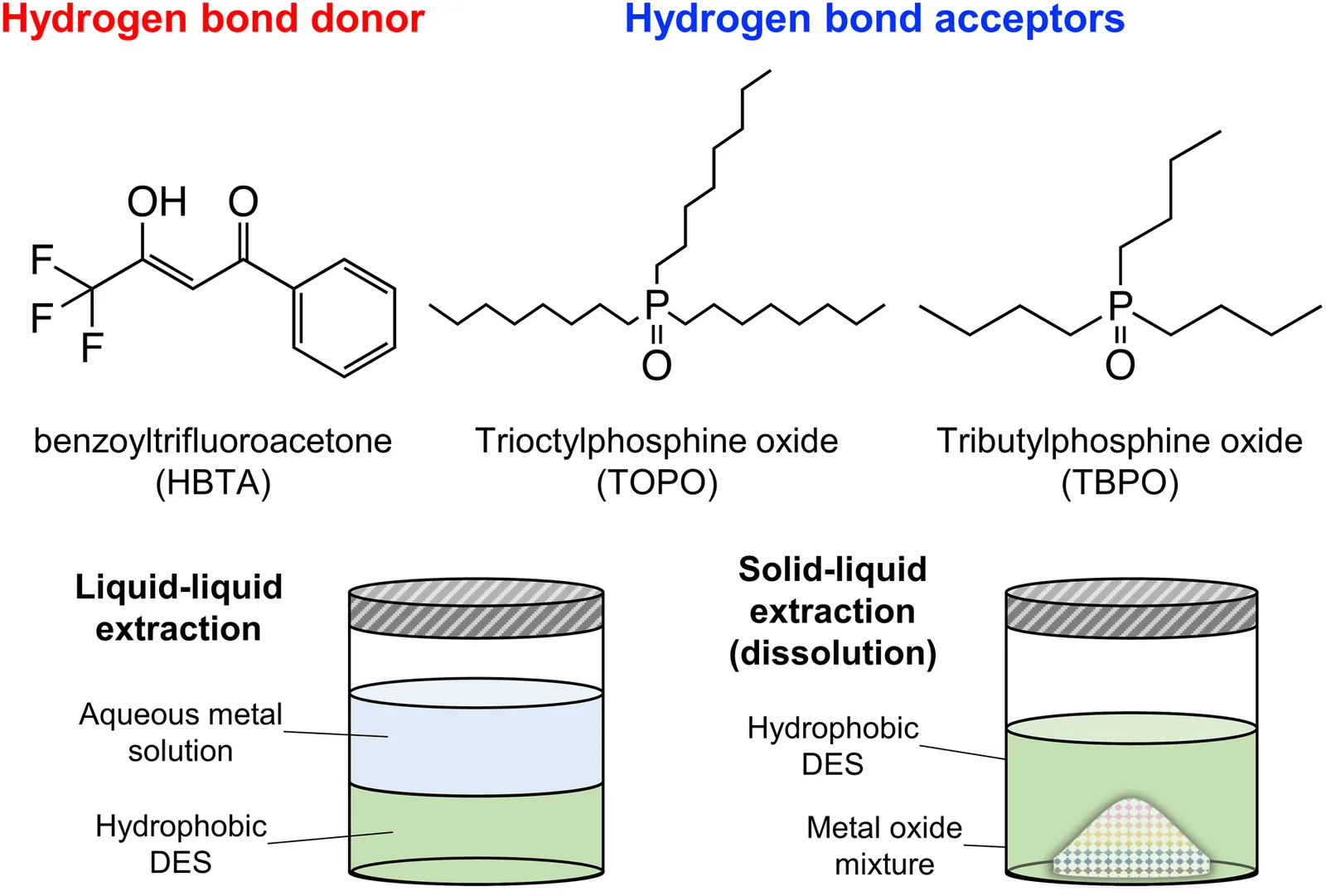
Chemical structures of the DES components and the schematics of the liquid–liquid extraction and solid–liquid extraction (dissolution) of metals using hydrophobic DESs.

The synergistic cooperation of HBTA and TOPO in the coordination of metals was verified by observation of the liquid–liquid extraction behaviors (Fig. S1†). Using single ligand systems employing individual HBTA or TOPO no extraction of Nd(iii), Dy(iii), and Co(ii) was observed in the studied pH range from 1 to 3, indicating unsatisfactory affinity of the individual ligands toward these metals. Only Fe(iii) was extracted gradually by the single HBTA system with an increase in pH. Using a mixed ligand system with HBTA and TOPO different tendencies during extraction were observed; not only Fe(iii) but also Nd(iii) and Dy(iii) were efficiently extracted even at pH below 1, and Co(ii) extraction was gradually increased with an increase in pH above 2. Therefore, HBTA and TOPO performed synergistically in the coordination of Fe(iii), Nd(iii), Dy(iii), and Co(ii). Subsequently, extraction experiments were performed to investigate the most favorable ligand ratio for the extraction of Nd(iii) and Dy(iii) (Fig. S2†). There was a slight difference in the optimal composition of ligands for the extraction of Nd(iii) and Dy(iii) in different systems. However, there is no significant difference in Nd/Dy separation at *X*_HBTA_ = 0.7, that is close to DES composition studied.

The liquid–liquid extraction behavior of metals with the DESs HBTA/TOPO and HBTA/TBPO were studied. The extraction performance of the DESs was similar for Nd(iii), Dy(iii), and Co(ii), while HBTA/TBPO extracted Fe(iii) in a slightly lower pH region than HBTA/TOPO (Fig. 2a and b). As expected, due to the greater charge density of Dy^3+^ over Nd^3+^, the former is more efficiently extracted at lower pH values in both studied systems. Additionally, a good selectivity between the trivalent and divalent metal was obtained in HBTA/TBPO at pH_eq_ = 1.6, with separation factors (SFs) of 785 and 312 obtained for the Dy/Co and Nd/Co pairs, respectively. However, SF for Dy/Nd was only 4.0 and Fe was poorly separated. Therefore, we hypothesized that both DESs had a similarly high affinity to the studied metals owing to the synergistic effect of the beta-diketone and the phosphine oxide ligands. In traditional solvent extraction system, synergistic cooperation of the mixed ligands can be modeled by the formation of aggregates through self-assembly resulting from the amphiphilic nature of the ligands, where the diversity of energetically similar aggregates entropically enhances extraction.^46^ Because the steric effect of ligands, which are expected to be more pronounced in DES due to the absence of diluent, has a significant influence on the diversity of the aggregates, it can influence the extraction and dissolution selectivity.

**Fig. 2:**
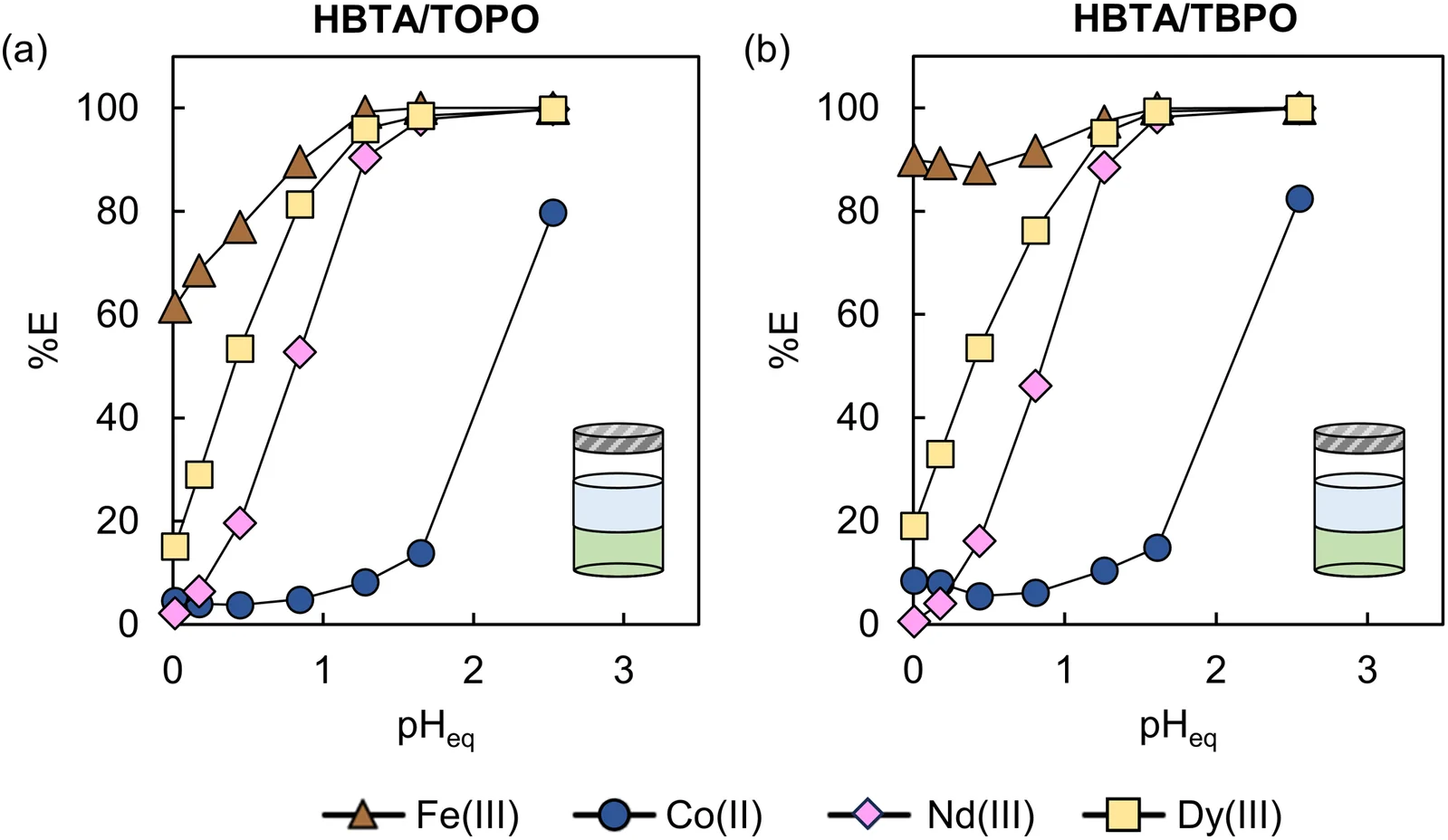
Liquid–liquid extraction efficiency (%*E*) of Fe(iii), Co(ii), Nd(ii), and Dy(iii) as a function of pH, (a) HBTA/TOPO (2 : 1) and (b) HBTA/TBPO (2 : 1) as the extraction phase, respectively. Experimental condition: aqueous phase 1 mmol L^−1^ metals in 1 mol L^−1^ (NH_4_)_2_SO_4_, A/O = 5 : 1, vigorously mixed at RT for 1 h.

The dissolution behavior of metal oxides, namely, Nd_2_O_3_, Dy_2_O_3_, CoO, and Fe_2_O_3_ into HBTA/TOPO and HBTA/TBPO, was investigated. It was hypothesized that the weak nature of the HBTA acid in non-aqueous media could be harnessed to increase the dissolution selectivity. A study on the effect of water on metal oxide dissolution efficiency into HBTA/TOPO showed that no metal dissolution was observed without the addition of water (Fig. S3†). An increase in the water content to 2.5 wt% yielded and improved metal oxide dissolution efficiency. The critical role of water in the dissolution and speciation of rare earth metals in non-aqueous leaching media was reported previously.^14^ The added water facilitated the acid deprotonation and acted as a ligand to metals and enhanced metal oxide dissolution.^47^ The ^31^P-NMR spectra of HBTA/TOPO and HBTA/TBPO with and without water indicated that water addition makes the system slightly more acidic, proved by the peak shift toward a lower magnetic field, to the same degree in both HBTA/TOPO and HBTA/TBPO (Fig. S4†). The FT-IR spectra of the DESs with and without water indicated that water has no significant impact on hydrogen bonding interactions between HBDs and HBAs (Fig. S5†). Therefore, the impact of water addition was almost equivalent on these two DESs.

The kinetics of Fe, Co, Nd, and Dy oxide dissolution efficiencies into HBTA/TOPO and HBTA/TBPO with water addition are shown in Fig. 3a and b. In HBTA/TOPO, the quantitative Nd dissolution was observed at 3 h. Interestingly, the dissolutions of Co and Dy oxides at 24 h were 8.8% and 2.7%, respectively. Contrary to the liquid–liquid extraction tests, application of the same solvents for dissolution showed an inversed preference for the lighter rare earth. Fe oxide dissolution was negligible, even after 24 h. The higher lattice energy of Fe oxide compared to Nd and Dy oxides, derived from much smaller ionic radii of Fe, makes Fe dissolution more difficult. In contrast, the dissolutions of Nd, Dy, and Co oxides increased with time, and quantitative Nd and Dy oxide dissolution was observed in HBTA/TBPO, while Fe oxide was only slightly dissolved. Considering that the DESs presented similarly high metal–ligand affinities with different aggregation stoichiometries, according to our liquid–liquid extraction studies, the dissolution selectivity of metal oxides into hydrophobic DESs is likely dominated by DES steric environment affecting the aggregation structures. The dissolution of heavy rare earth oxides, such as Dy oxide, has been challenging in previously reported DESs. However, HBTA/TBPO allows efficient dissolution of both Nd and Dy oxides, exhibiting great potential as a selective dissolution medium for heavy rare earths. The dissolution selectivity of rare earth oxides into DESs was studied previously. Brønsted acidic DESs were capable of dissolving light rare earth oxides, while heavy rare earth oxides, such as Dy, were poorly dissolved.^16^ Similarly, guanidine-based DESs were favorable for dissolving light rare earths over heavy rare earth oxides. The separation factor for Nd/Dy was 16.9 in their mixed system.^18^ The DESs were also reported for the selective dissolution of Nd over Fe oxide with the SF_Nd/Fe_ of more than 1300.^17^

**Fig. 3:**
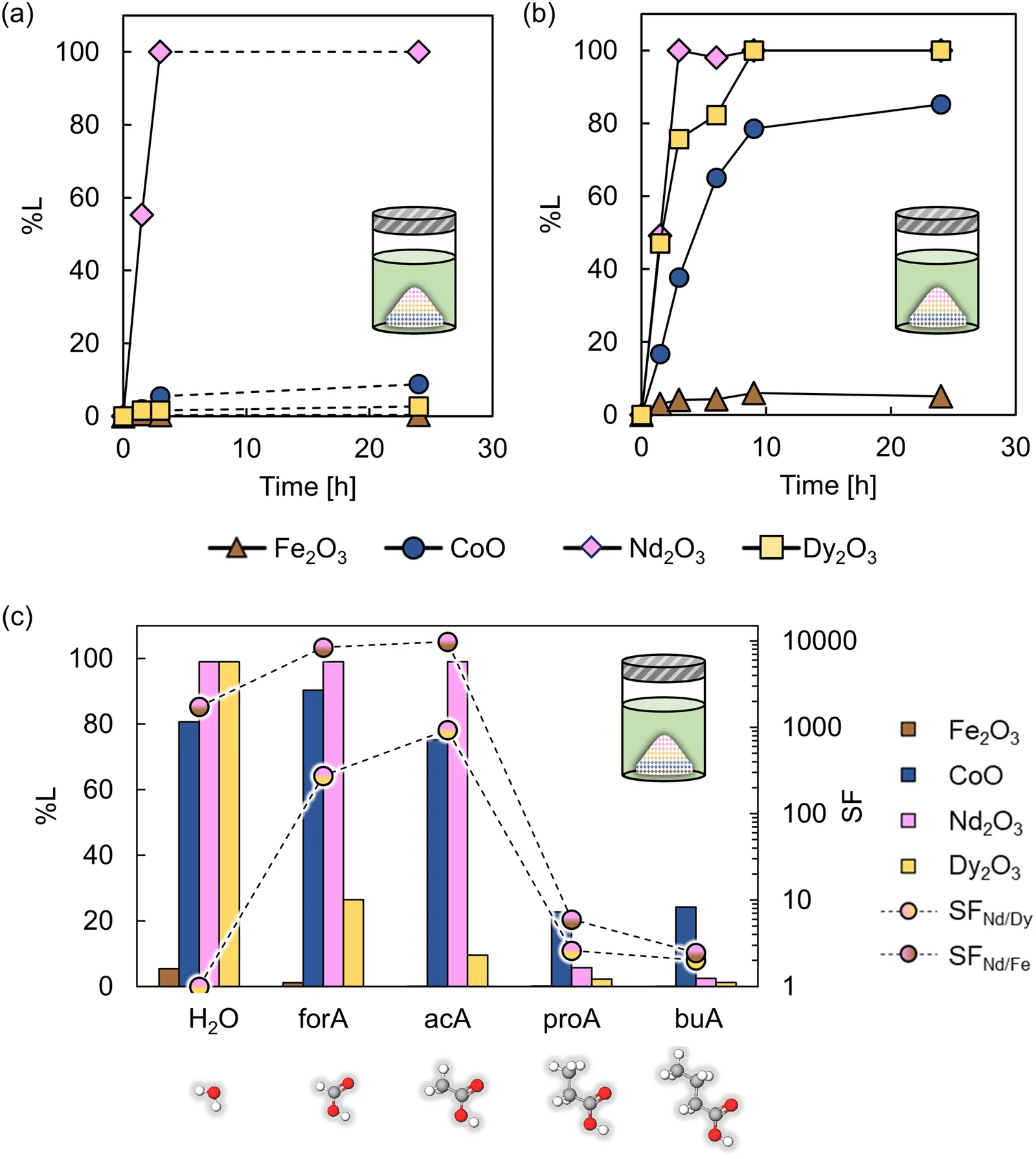
Kinetics of the dissolution of metal oxides into (a) HBTA/TOPO and (b) HBTA/TBPO with 2.5% H_2_O. Experimental condition: metal oxides to DES ratio of 5 g-each per L, stirring at 333 K at 400 rpm. (c) Dissolution efficiency (%*L*) of metal oxides into HBTA/TBPO with H_2_O or carboxylic acids with different alkyl chain lengths. Experimental condition: metal oxides to DES ratio of 5 g-each per L, stirring at 333 K at 400 rpm for 24 h, additive concentration of 1 mol L^−1^ in the DES.

The effects of adding water, amphiphiles, and even apolar molecules into DESs have been extensively studied. Importantly, the addition of such molecules into DESs causes changes in the DES nanostructure.^48–50^ Therefore, a modification of the DES steric environment was attempted using the addition of carboxylic acids of varying alkyl chain lengths as water analogs, which can bond hydrogen and donate protons. Here, formic acid (forA), acetic acid (acA), propionic acid (proA), and butyric acid (buA) were employed at a concentration of 1 mol L^−1^ in HBTA/TBPO, respectively. The dissolution efficiencies of metal oxides into HBTA/TBPO with water or carboxylic acids are shown in Fig. 3c. The DES with water allowed Co, Nd, and Dy oxide dissolution. The addition of forA or acA was similar to that of water in terms of the dissolution efficiency of Co and Nd oxides. In contrast, the Fe and Dy oxides dissolution gradually decreased with an increase in the carbon number of the carboxylic acid. The DESs with proA and buA significantly suppressed Nd and Dy oxide dissolution. There was a clear association between an increased carbon number of the added carboxylic acid and suppression of Co and Dy oxide dissolution. The SFs for Nd/Dy and Nd/Fe were significantly improved from 1 and 1740 to 936 and 9800, respectively, when additives were changed from H_2_O to acA into HBTA/TBPO. The p*K*_a_ values of forA, acA, proA, and buA are 3.75, 4.76, 4.88, and 4.82, respectively. There is no significant gap in p*K*_a_ values among the studied carboxylic acid additives. Therefore, it was suggested that the steric effects of additives influence the leaching selectivity, as well as the DES structure.

Perfluoroalkyl substances, such as HBTA, have suffered from environmental persistence due to the strong C–F bond, making the compounds hardly degradable. Therefore, a greener alternative, benzoylacetone, was employed as the HBD to demonstrate a more environmentally harmonious system. As shown in Fig. S6,† although the leaching efficiency decreased without the trifluoromethyl group, the selective dissolution of Nd was successfully performed. Therefore, a variety of greener beta-diketone ligands present a potential option to improve the dissolution efficiency and selectivity.

Finally, the reusability of the hydrophobic DES for the selective dissolution of metal oxides was demonstrated. The experimental details are described in the ESI.† As shown in Fig. 4, after the selective dissolution, stripping, and scrubbing of the DES phase, the DES could be successfully reused for selective dissolution, maintaining both its dissolution efficiency and selectivity.

**Fig. 4:**
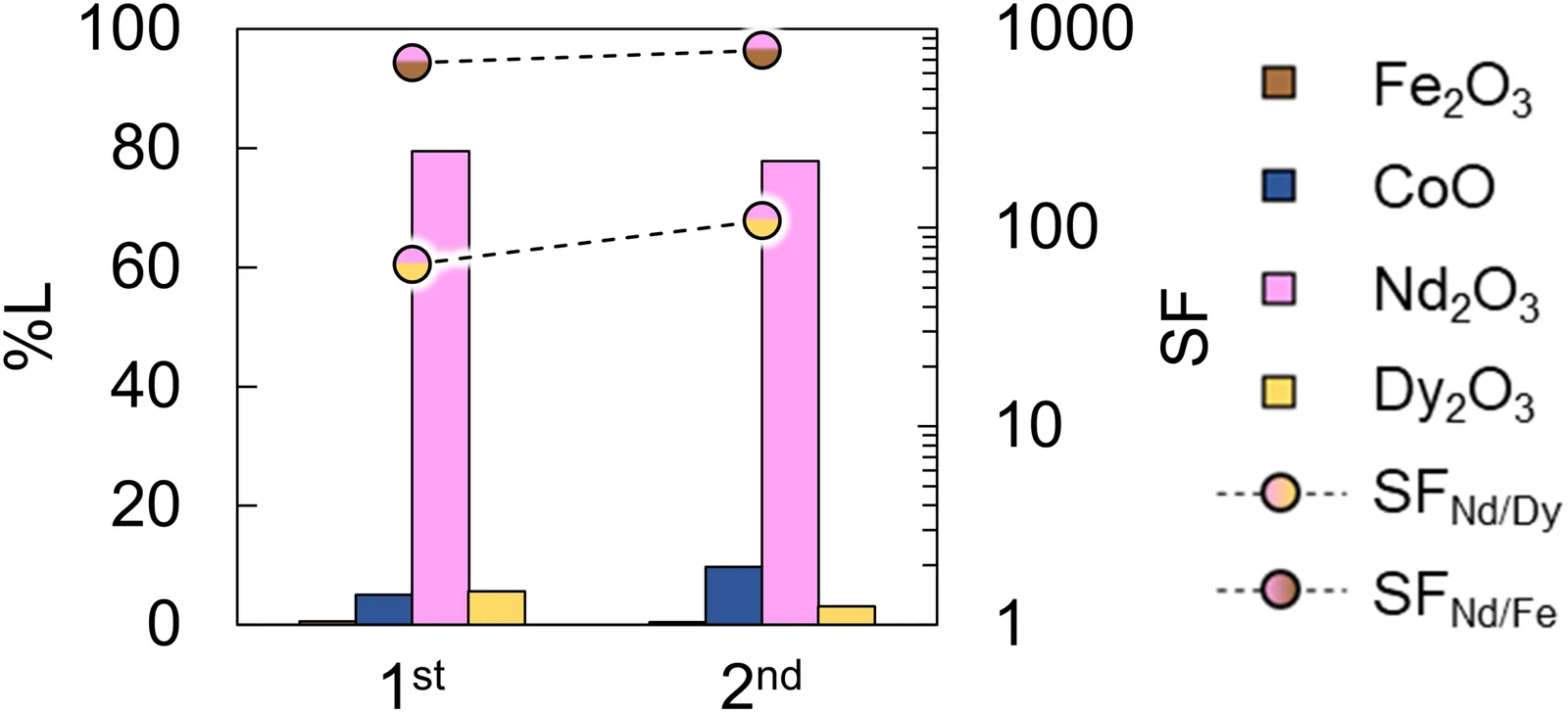
Reusability performance for the selective dissolution of metal oxides into HBTA/TOPO with acA system. Dissolution condition: metal oxides to DES ratio of 5 g-each per L, stirring at 333 K at 400 rpm for 3 h, acA concentration of 1 mol L^−1^ in the DES. The details of the stripping and scrubbing procedures are described in ESI.†

In this study, the liquid–liquid extraction and dissolution behavior of metals into hydrophobic DESs involved in HBAs having different alkyl chain lengths, namely, HBTA/TOPO and HBTA/TBPO, was investigated. The liquid–liquid extraction experiments showed that HBTA/TOPO and HBTA/TBPO have a similarly high affinity to the studied metal ions, and these metals were poorly separated. In dissolution experiments, the dissolution selectivity of the metal oxides into DESs differed significantly. HBTA/TOPO provided a selective Nd oxide dissolution, while HBTA/TBPO allowed Co, Nd, and Dy oxide dissolution. Moreover, the dissolution selectivity of Co, Nd, and Dy oxides into HBTA/TBPO could be controlled by the addition of the carboxylic acids with different alkyl chain lengths. These results suggest that the steric environment of DES is a key factor for improving the dissolution selectivity of metal oxides including the traditionally difficult rare earths. The maximum separation factors achieved for Nd/Dy and Nd/Fe in HBTA/TBPO with acA were notably high, reaching 936 and 9800, respectively, which, to the best of our knowledge, are the highest reported values so far. Finally, the DES could be reused for further selective dissolution operation owing to its hydrophobic feature. Overall, expanding the application of hydrophobic DESs from extraction to dissolution media enabled more selective recovery of rare earth elements. The dissolution selectivity could be improved by the molecular design of DESs and additives, realizing a greener separation process without the emission of harmful acids and organic solvents. This research contributes to the Sustainable Development Goals 12 and 13, “responsible consumption and production” and “climate action”.

## Conflicts of interest

There are no conflicts of interest to declare.

## Supplementary Material

GC-026-D4GC01089B-s001

## Data Availability

The data supporting this article have been included as part of the ESI.†
