# Supplementary material for: Improved separation of rare earth elements using hydrophobic deep eutectic solvents: liquid–liquid extraction to selective dissolution
Source: Green Chem. 2024 Jul 11;26(18):9671–5. doi: 10.1039/d4gc01089b (PMC13725688; doi:10.1039/d4gc01089b)
Supplement: Supplementary file 1 [file GC-026-D4GC01089B-s001.pdf]

Electronic Supplementary Material (ESI) for Green Chemistry.
This journal is © The Royal Society of Chemistry 2024

Supporting information

Improved separation of rare earth elements using hydrophobic deep eutectic solvents: liquid-liquid extraction to selective dissolution

Takafumi Hanada[a,b*], Nicolas Schaeffer[b], Masahiro Katoh[a], Joao A. P. Coutinho[b], Masahiro Goto[c*]

[a] *Department of Applied Chemistry, Graduate School of Technology, Industrial and Social Sciences, Tokushima University, 2-1 Minamijosanjima, Tokushima, Japan*

[b] *CICECO-Aveiro Institute of Materials, Department of Chemistry, University of Aveiro, 3810-193 Aveiro, Portugal*

[c] *Department of Applied Chemistry, Graduate School of Engineering, Kyushu University, 744 Motooka, Fukuoka, Japan*

*Corresponding authors:

Takafumi Hanada hanada@tokushima-u.ac.jp

Masahiro Goto m-goto@mail.cstm.kyushu-u.ac.jp

Experimental

1. Reagents and chemicals: benzoyltrifluroacetone (HBTA), benzoylacetone (HBzAc), tributylphosphine oxide (TBPO), and trioctylphosphine oxide (TOPO) were purchased from Tokyo Chemical Industry Co., Ltd. Iron(III) oxide, cobalt(II) oxide, neodymium(III) oxide, and dysprosium(III) oxide, chloroform, ammonium sulfate, sulfuric acid, metal standard solutions (1000 ppm) of iron, cobalt, neodymium, and dysprosium were purchased from FUJIFILM Wako Pure Chemical Co., Ltd. All the reagents were used as received. Milli-Q water (Milli-Q Integral 3, Merck Millipore). was used to prepare all the aqueous solutions.

2. Preparation and characterization DESs: The DESs were prepared according to our previous study.[1] In short, a hydrogen bond donor (HBD) and a hydrogen bond acceptor (HBA) are weighted with a targeted molar ratio and stirred under 333 K until homogenized. The density and viscosity of DESs were measured (**Table S1**). The intermolecular interaction between HBD and HBA were characterized by Fourier-transformed infra-red spectroscopy (FT-IR, Spectrum Two, Perkin-Elmer) and nuclear magnetic resonance spectroscopy (NMR, ECZ400S, JEOL).

3. Liquid-liquid extraction of metal ions: The aqueous phase was prepared as 1 mmol-each/L mixed metal solution containing 0.1 or 1 mol/L ammonium sulfate. The initial pH was adjusted by concentrated sulfuric acid. For traditional solvent extraction, the organic phases were prepared by dissolving individual or mixed ligands in chloroform with known concentrations. Also, the DESs were directly used as the organic phase. The aqueous and organic phases were contacted at the aqueous:organic (A/O) volume ratio of 5:1, and were vigorously mixed under ambient temperature for 1 h. After extraction, the aqueous phase was separated by centrifugation, and the metal content was evaluated by inductively coupled plasma optical

emission spectrometer (ICP-OES, optima 8300, Perkin-Elmer). The extraction efficiency (%E), distribution ratio (D), and separation factor (SF) were evaluated as follows,

$$\%\mathrm{E} = \frac{C_{init} - C_{aq}}{C_{init}} \times 100 \qquad \mathrm{D} = \frac{C_{init} - C_{aq}}{C_{aq}} \qquad \mathrm{SF_{A/B}} = \frac{\mathrm{D_A}}{\mathrm{D_B}}$$

where C_{init} and C_{aq} were the concentration of a metal in the initial and the equilibrated aqueous phase, respectively.

4. Dissolution of metal oxides into DESs: The DESs with water or water-like additives were prepared by adding the additives to the DES and stirring at 400 rpm at 333 K for 1 h to make the mixture homogeneous. This pre-treated DES was added to the mixture of iron(III) oxide, cobalt(II) oxide, neodymium(III) oxide, and dysprosium(III) oxide with the weight ratio of 5 mg-each/mL (20mg-total/mL). The mixture was reacted under 333 K stirring at 400 rpm for 24 h. The reaction mixture was centrifuged to recover the DES phase from the residue. The metal content in the DES phase was evaluated by ICP-OES and total reflection x-ray fluorescence spectrometer (TXRF, picofox x2, Bruker). The detail in the analytical method was reported previously.[1] The dissolution efficiency (%L), stripping efficiency (%S), and SF in dissolution were evaluated as follows,

$$\%\mathrm{L} = \frac{C_{dissol} \times \mathrm{V_{DES}}}{m_{total}} \times 100 \qquad \%\mathrm{S} = \frac{C_{dissol} - C_{strp}}{C_{dissol}} \times 100 \qquad \mathrm{SF_{A/B}} = \frac{\mathrm{L_A}/(1 - \mathrm{L_A})}{\mathrm{L_B}/(1 - \mathrm{L_B})}$$

where C_{dissol}, C_{strp}, $\mathrm{V_{DES}}$, and m_{total} were the concentration of the dissolved metal in DES, concentration of metal in DES after stripping, the volume of the DES, and the total molar amount of the metal, respectively. Considering the detection limit of our ICP system, the values of 0.99 and 0.01 for L were used in case of quantitative (mainly Nd) and negligible (mainly Fe) dissolution.

Table S1. Density and viscosity of DESs with and without the addition of water. The water contents, densities, and viscosities of the DESs were measured using a Karl Fischer coulometric titrator KF200 (Mitsubishi Chemical Analytech, Japan), a density meter DMA 35 Ampere (Anton Paar, Austria), and a viscosity meter Lovis 2000M (Anton Paar, Austria), respectively.

	Water content [%]	Density [g/cm^3]	Viscosity [g/cm^3]
HBTA/TOPO(2:1)	0.28	1.05	13.3
	2.48	0.96	28.9
HBTA/TBPO(2:1)	0.13	1.13	6.3
	2.15	1.15	15.7

- Traditional liquid-liquid extraction study.

The liquid-liquid extraction of lanthanides with a beta-diketone and a phosphine oxide was known as a representative synergistic extraction system.[2] The extraction equation is generally described as follows:

$$3HL_{org} + Ln^{3+}_{aq} + nS_{org} \rightleftarrows [LnL_3S_n]_{org} + 3H^{+}_{aq}$$

where HL, Ln, S represents the diketone ligand, lanthanide ion, and phosphine oxide co-ligand, respectively.

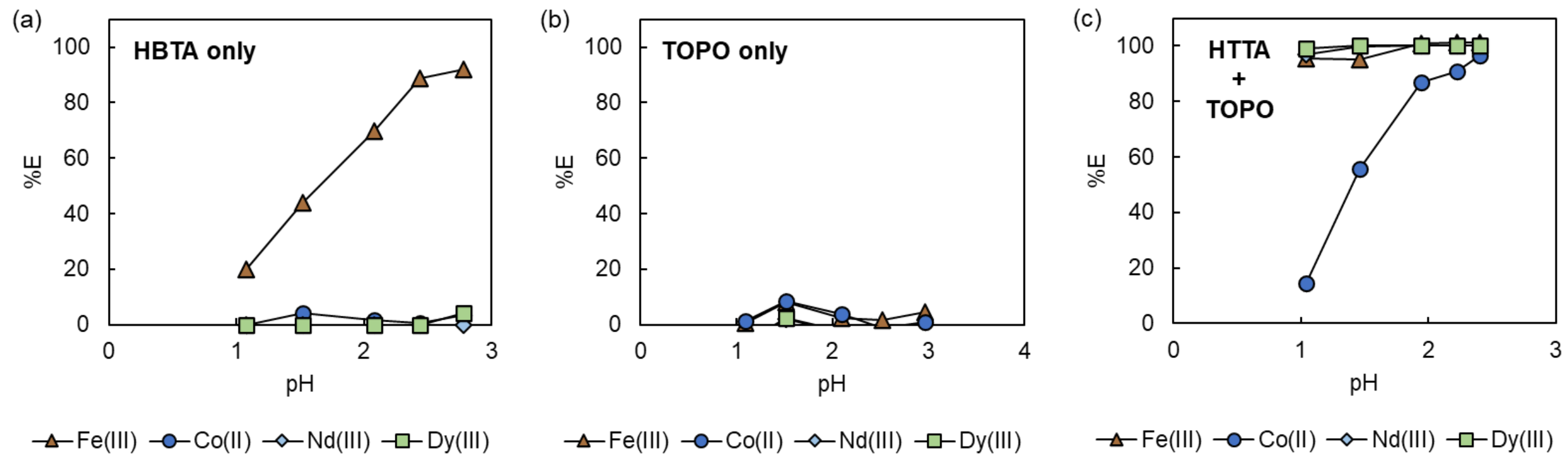

Fig. S1. Comparison of the Liquid-liquid extraction behavior of Fe(III), Co(II), Nd(III), and Dy(III) with conventional solvent extraction with chloroform as diluent and the DES-based extraction without diluent. Experimental condition: [ligand]=1 mol/L for individual ligand systems and [HTTA]=[TOPO]=0.5 mol/L in mixed ligand system.

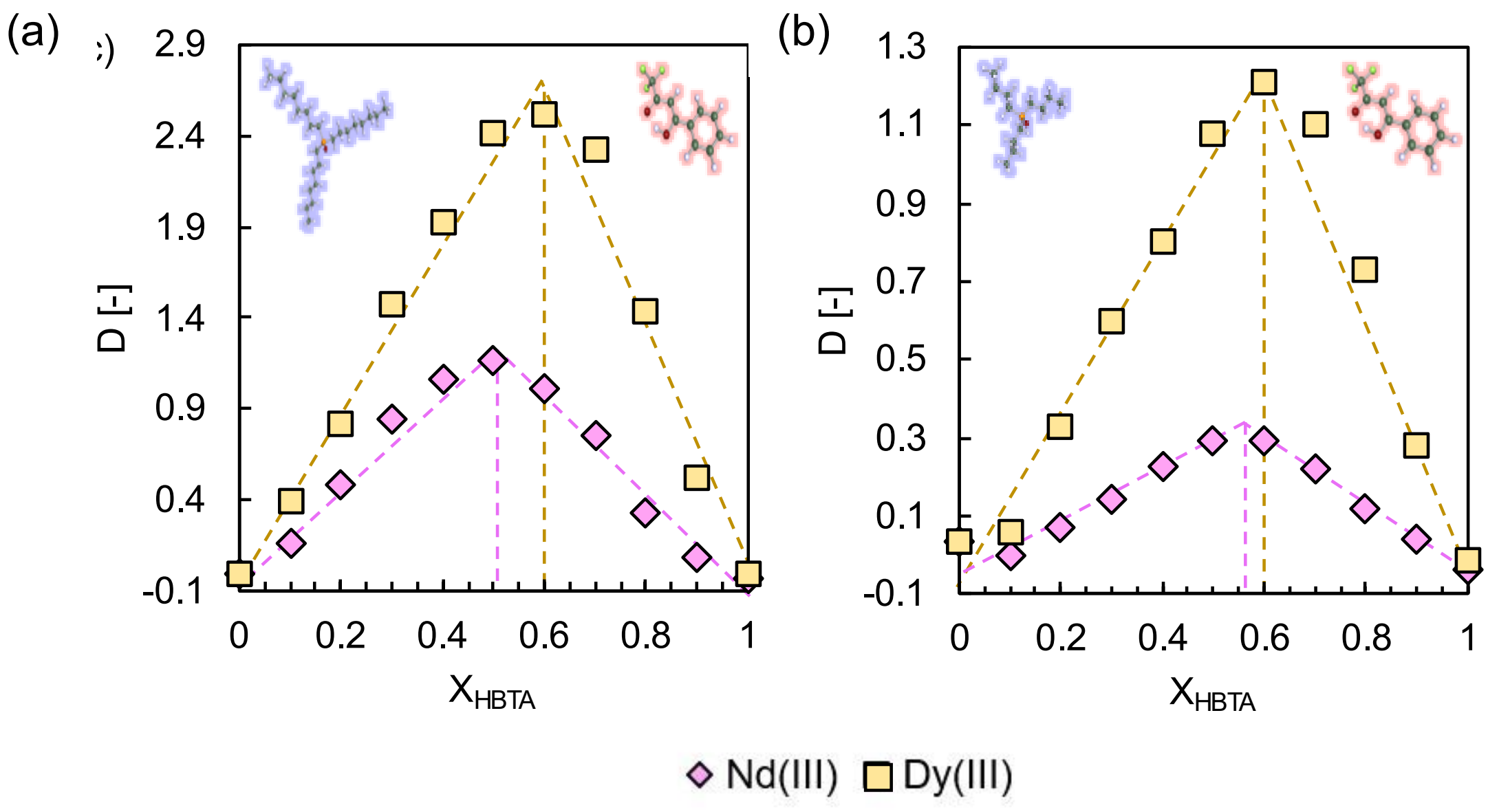

Fig. S2. Distribution ratio (D) of Nd(III) and Dy(III) in (a) HBTA/TOPO and (b) HBTA/TBPO with different molar ratios of HBTA to HBAs (X_{HBTA}). Experimental condition: 1 mmol/L metal in 1 mol/L $(NH_4)_2SO_4$ as the aqueous phase, [HBTA] + [TOPO or TBPO] = 1 mol/L dissolved in chloroform as the organic phase, contacting aqueous and organic phases at a volume ratio of 1:1, vigorously mixed for 1 h at RT.

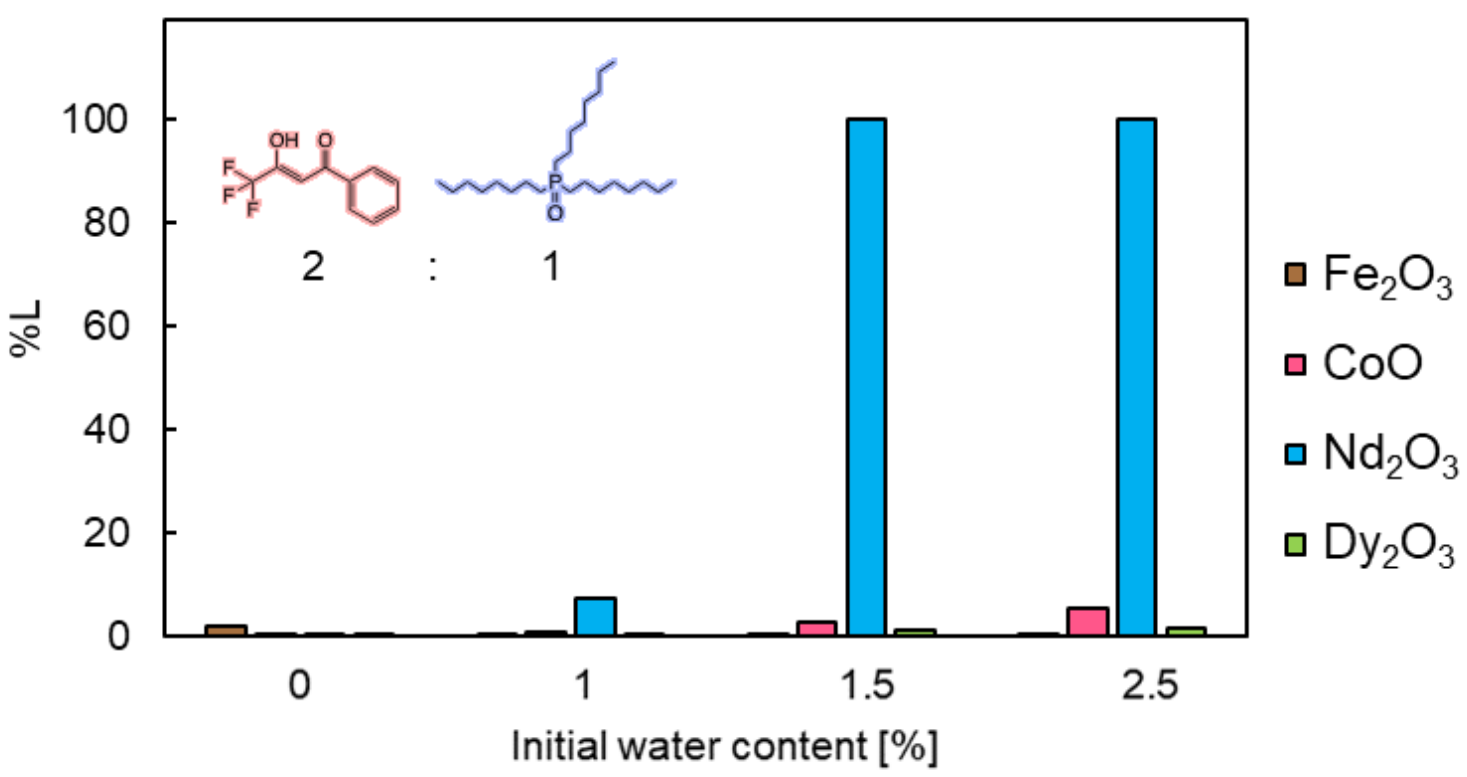

Fig. S3. Dissolution of iron(III), cobalt(II), neodymium(III), and dysprosium(III) oxides into HBTA/TOPO(2:1) as a function of water content. Experimental conditions: 5 mg/mL of each metal oxides, stirring at 333 K at 400 rpm.

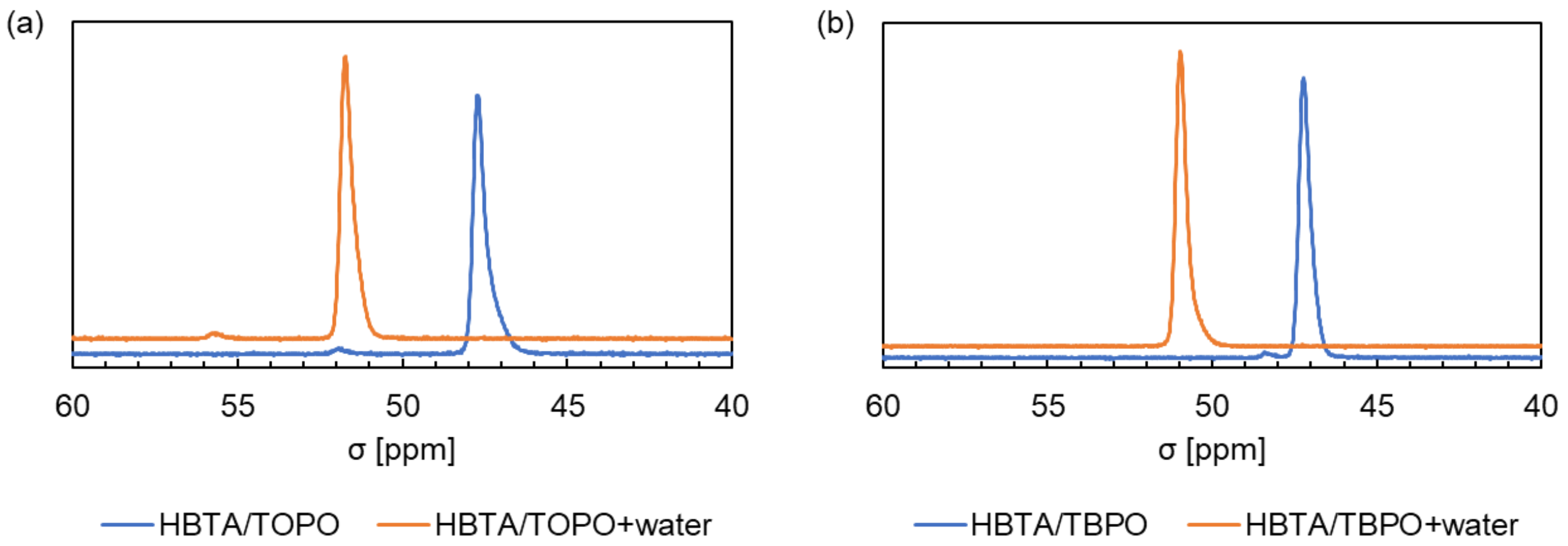

Fig. S4. ^{31}P-NMR spectra of HBTA/TOPO(2:1) and HBTA/TBPO(2:1) without or with the addition of 2.5% of water. The NMR samples were prepared using co-axial insert containing H_2PO_4 in D_2O for inner insert, and were measured by a NMR spectrometer ECZ400S (JEOL, Japan).

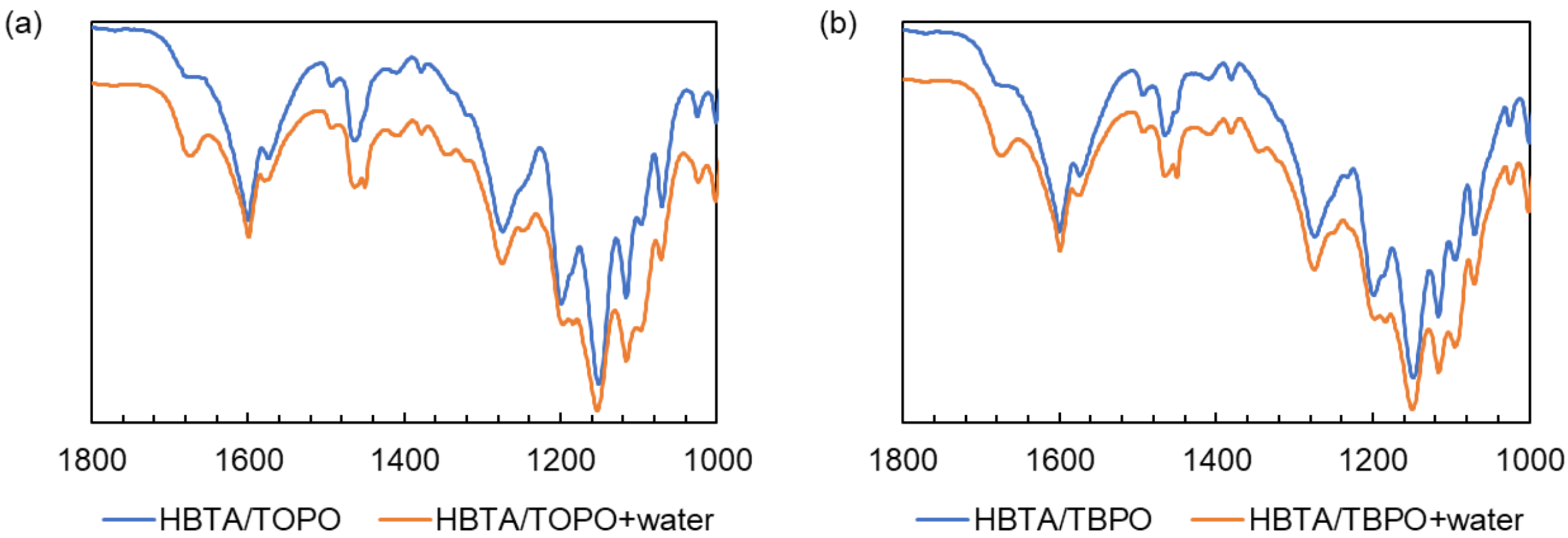

Fig. S5. FT-IR spectra of HBTA/TOPO(2:1) and HBTA/TBPO(2:1) without or with the addition of 2.5% of water. The FT-IR spectra were measured by ATR-FTIR spectrometer Spectrum two (Perkin-Elmer, USA).

- Selective dissolution with greener beta-diketone ligands

The DES composed of an non-fluorinated analog of HBTA, namely, Benzoyltrifluoroacetone (HBzAc) was prepared with TOPO as the HBA with the molar ratio of 2:1. The dissolution behavior of metal oxides was compared with HBTA/TOPO (**Fig. S6**).

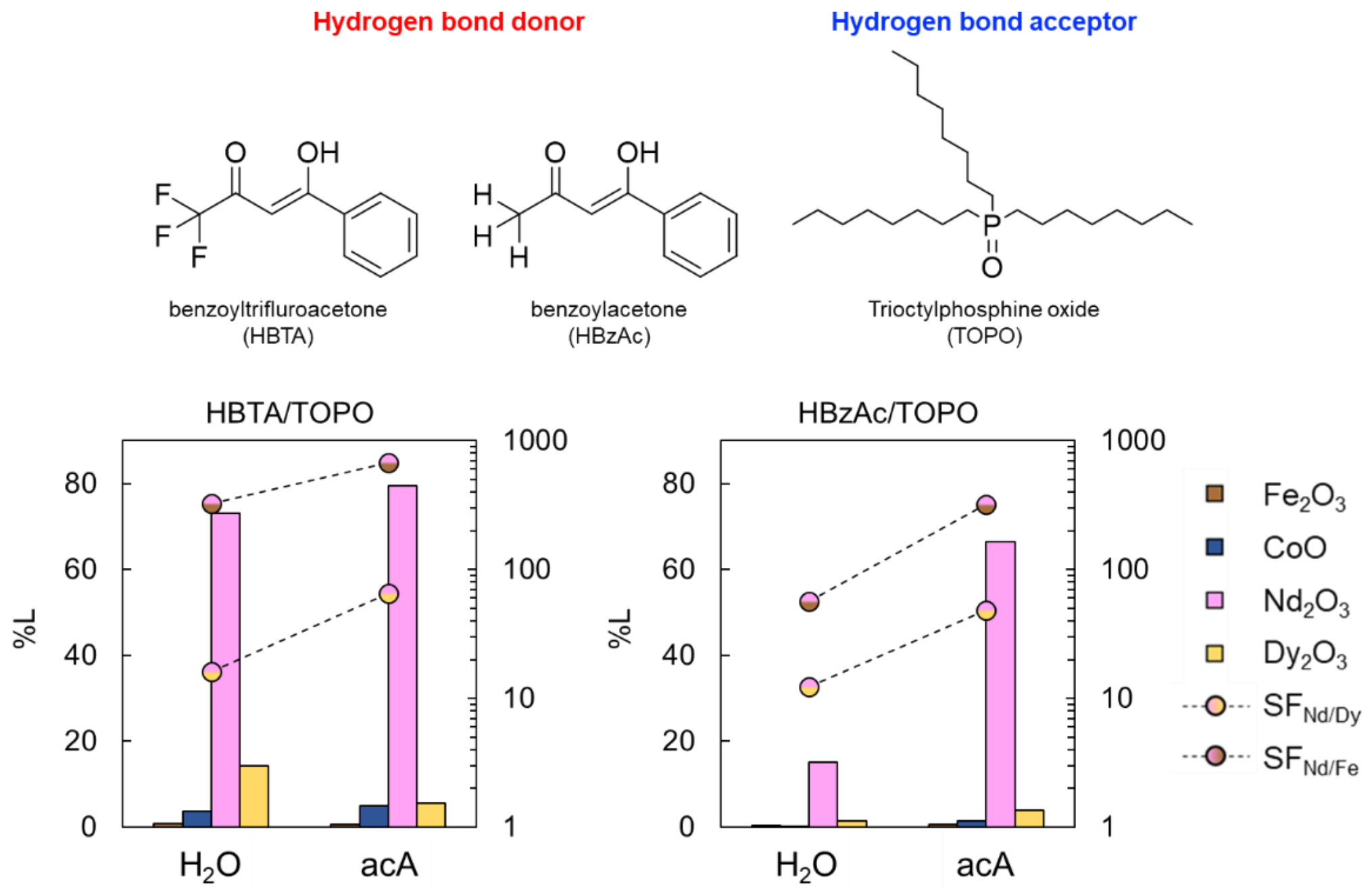

Fig. S6. Dissolution behavior of metal oxides with HBDs with and without perfluoro- functional group. Experimental condition: metal oxides to DES ratio of 5 g-each/L, 1 mol/L additives, stirring at 333 K at 400 rpm for 3 h.

- Reusability study

Several steps were needed to reuse the DES after the selective dissolution. The 1st dissolution was performed by HBTA/TOPO with acA system as an example. The acidic aqueous solutions could be used for stripping dissolved metals from the DES. The stripping performance of each metal by oxalic acid and sulfuric acid is shown in **Fig. S7**. After stripping, the DES couldn't directly be reused for the further selective dissolution step due to the contamination of the DES by acA, H_2O, and acids. For stripping, 3 mol/L sulfuric acid was used for further reusability investigation to prevent the acid contamination. Moreover, the DES

phase was washed with 0.01 mol/L NaOH and pure water to completely remove acidic contaminants. The DES phase was dried at 373 K for 0.5 h for dehydration. Then the refreshed DES was used for the 2nd leaching with the addition of acA. The leaching and stripping performances of the reusability test is shown in **Fig. 4**. The leaching selectivity and efficiency of the studied metal oxides were similarly high even for the regenerated DES.

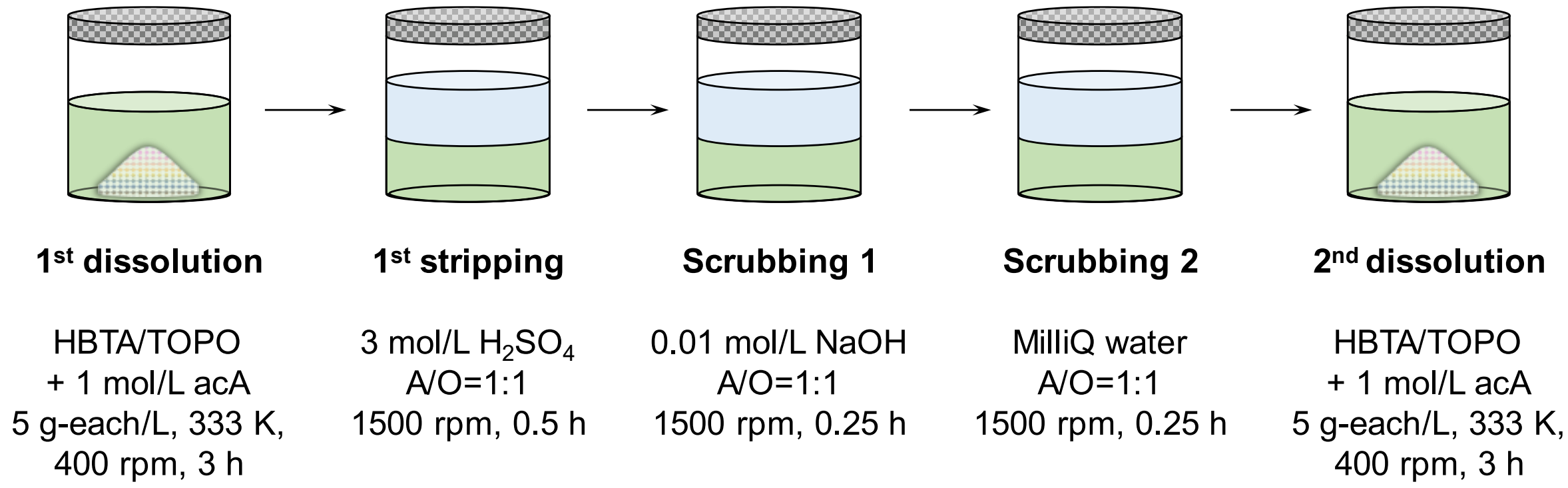

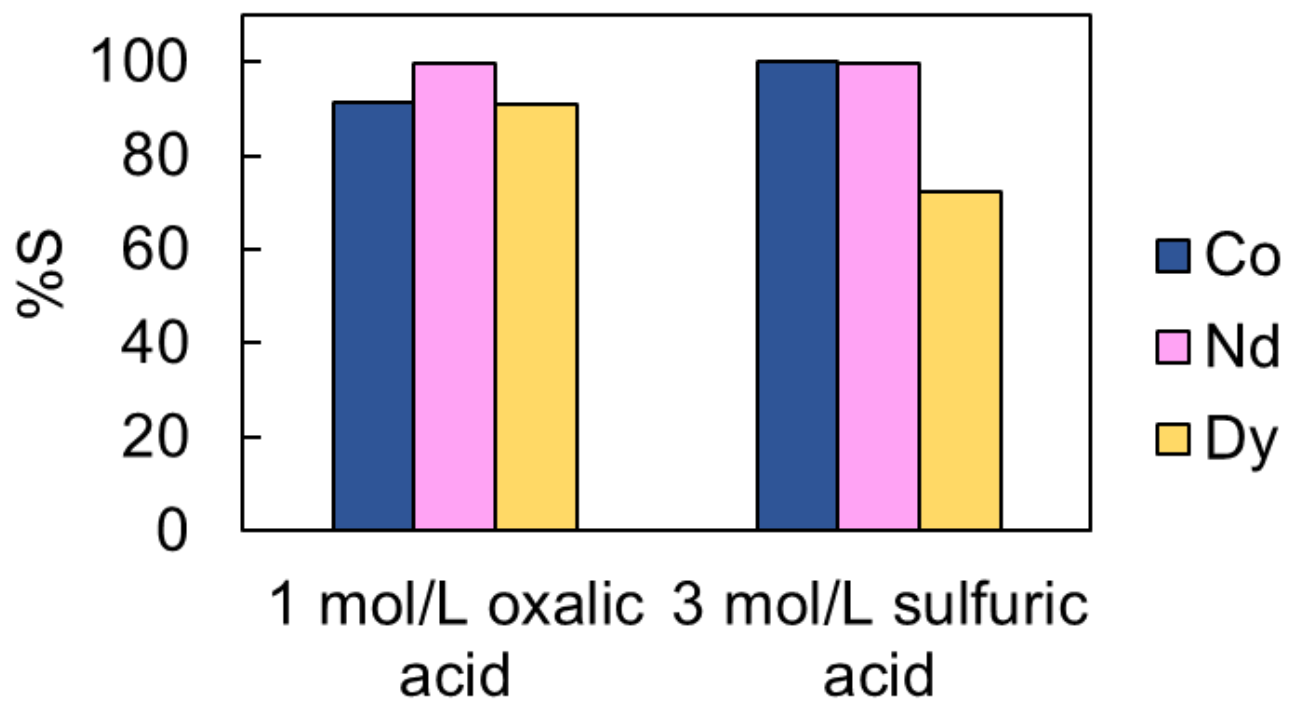

Fig. S7. Stripping of dissolved metals from metal-loaded HBTA/TOPO. Experimental condition: A/O=1:1, mixing at 1500 rpm for 0.5 h at RT.

- Data reproducibility

Table S2. Dissolution efficiency of metal oxides into HBTA/TOPO with the addition of 1 mol/L H_2O or acetic acid (acA). Experimental condition: metal oxides to DES ratio of 5 g-each/L, stirring at 333 K at 400 rpm for 3 h. SD represents standard deviation (n=3).

		Dissolution efficeincy (%L)			
	n	Fe	Co	Nd	Dy
HBTA/TOPO + 1 mol/L H_2O	1	0.93	2.43	74.91	14.10
	2	0.54	4.09	70.87	9.10
	3	1.01	4.83	73.36	19.65
	average	0.83	3.78	73.05	14.28
	SD	0.3	1.2	2.0	5.3
HBTA/TOPO + 1 mol/L acA	1	0.56	4.77	77.71	5.76
	2	0.38	4.55	80.36	5.36
	3	0.78	5.74	80.50	5.75
	average	0.57	5.02	79.53	5.63
	SD	0.2	0.6	1.6	0.2

References

1 T. Hanada and M. Goto, *Green Chemistry*, 2022, **24**, 5107–5115.

2 M. Atanassova, *Separations*, 2022, **9**, 154.
